# Angiopoietin1 Inhibits Mast Cell Activation and Protects against Anaphylaxis

**DOI:** 10.1371/journal.pone.0089148

**Published:** 2014-02-19

**Authors:** Jun-Hua Yao, Ming Cui, Meng-Tao Li, Yi-Nan Liu, Qi-Hua He, Jun-Jun Xiao, Yun Bai

**Affiliations:** 1 Department of Cell Biology, School of Basic Medical Sciences, Peking University Health Science Center, Beijing, China; 2 Department of Cardiology, Peking University Third Hospital, Beijing, China; Juntendo University School of Medicine, Japan

## Abstract

Since morbidity and mortality rates of anaphylaxis diseases have been increasing year by year, how to prevent and manage these diseases effectively has become an important issue. Mast cells play a central regulatory role in allergic diseases. Angiopoietin1 (Ang-1) exhibits anti-inflammatory properties by inhibiting vascular permeability, leukocyte migration and cytokine production. However, Ang-1's function in mast cell activation and anaphylaxis diseases is unknown. The results of our study suggest that Ang-1 decreased lipopolysaccharide (LPS)-induced pro-inflammatory cytokines production of mast cells by suppressing IκB phosphorylation and NF-κB nuclear translocation. Ang-1 also strongly inhibited compound 48/80 induced and FcεRI-mediated mast cells degranulation by decreasing intracellular calcium levels *in vitro*. *In vivo* lentivirus-mediated delivery of Ang-1 in mice exhibited alleviated leakage in IgE-dependent passive cutaneous anaphylaxis (PCA). Furthermore, exogenous Ang-1 intervention treatment prevented mice from compound 48/80-induced mesentery mast cell degranulation, attenuated increases in pro-inflammatory cytokines, relieved lung injury, and improved survival in anaphylaxis shock. The results of our study reveal, for the first time, the important role of Ang-1 in the activation of mast cells, and identify a therapeutic effect of Ang-1 on anaphylaxis diseases.

## Introduction

When Angiopoietin1 (Ang-1) was first discovered as a specific ligand of Tie-2 in 1996, people were concerned about its role in promoting angiogenesis [1]. Ang-1 cooperates with vascular endothelial growth factor (VEGF) in the later stages of embryonic angiogenesis to form the mature vascular endothelial barrier [2]. Moreover, in adult microvasculature, binding of Ang-1 to the Tie-2 receptor stabilizes endothelial cell interactions with the extracellular matrix and junctional proteins, and enhances endothelial barrier functions [3]. Transgenic mice over-expressing Ang-1 in dermal micro-vessels were resistant to leakage of albumin-binding Evans blue dye in response to VEGF and other inflammatory agents [4]. Adenoviral-mediated delivery of Ang-1 in adult mouse vascular endothelia markedly reduced vascular leakage [5]. An improved mortality rate in mice with endotoxic shock was seen with an adenoviral construct encoding Ang-1 pretreatment [6]. Local administration of recombinant Ang-1 protects against histological, biochemical, and functional changes observed in an OVA-induced mouse allergic asthma model [7]. These findings raise the possibility that Ang-1 has anti-inflammatory properties. *In vitro* studies have found that Ang-1 directly stimulates migration, and possibly inhibits vascular endothelial growth factor-induced eosinophil and neutrophil chemotaxis [8], [9]. Moreover, Ang-1 can promote monocyte chemotaxis, endothelial binding, and trans-endothelial migration, which are key events in the progression of atherosclerosis [10]. The Ang-1/Tie-2 signaling pathway inhibits lipopolysaccharide (LPS)-induced activation of macrophage cells [11].

Mast cells are part of the innate immune system and participate in the first line of defense against pathogens, such as bacteria and parasites, and release granules after activation [12]. Traditionally, mast cells are considered major effectors in acute allergic reactions associated with urticaria, rhinitis, atopy, anaphylaxis, and in combination with chronic allergic inflammation [13]. The primary response to Toll-like receptor (TLR) ligands is the production of inflammatory cytokines, rather than degranulation [12]. Mast cell activation can be elicited by not only the aggregation of cell surface-specific receptor for IgE, FcεRI, but also the basic secretagogue, compound 48/80 [14]. Previous research has demonstrated that an appropriate concentration of compound 48/80 can activate mast cell exocytosis and cause anaphylaxis shock in animals [15].

Since morbidity and mortality rates of anaphylaxis diseases have been increasing year by year, how to prevent and manage these diseases effectively has become an important issue. We established mast cell activation models using LPS, compound 48/80 and FcεRI, respectively *in vitro* and IgE-dependent passive cutaneous anaphylaxis (PCA) and compound 48/80-induced anaphylaxis shock models *in vivo* to study the functions of Ang-1 in mast cell activation and anaphylaxis diseases. Our results suggested the important role of Ang-1 in the activation of mast cells, and have identified a therapeutic effect of Ang-1 on anaphylaxis diseases.

## Materials and Methods

### Cell lines and regents

The mouse mastocytoma cell line P815 was provided by National Platform of Experimental cell Resources for Sci-Tech (Beijing, China). P815 mast cells were maintained in Dulbecco modified Eagle's medium (Gibco, USA) supplemented with 10% fetal bovine serum (FBS, Gibco, USA), 100 IU/ml penicillin, and 100 µg/ml streptomycin in a humidified atmosphere of 5% CO2, 95% air at 37°C. RGDyK (RGD) was synthesized by Peptides International (Louisville, KY). Recombinant human Ang-1 and recombinant human Tie2/Fc chimera (soluble extracellular domain of tunica intima endothelial kinase 2 and Fc fusion protein, sTie-2) were purchased from R&D Systems (Minneapolis, MN, USA). A plasmid containing human Ang-1 cDNA was kindly provided by Regeneron Pharmaceuticals (Tarrytown, NK, USA).

### Mice

Specific pathogen-free male CD-1 mice were purchased from Charles River Laboratories (USA) and housed at 23°C±3°C under a 12-h light/dark cycle. All animal protocols used in this study were approved by the Committee of Animal Experiments of Peking University.

This study was carried out in strict accordance with the recommendations in the Guide for the Care and Use of Laboratory Animals of the National Institutes of Health. The protocol was approved by the Committee of Animal Experiments of Peking University. All surgery was performed under sodium pentobarbital anesthesia, and all efforts were made to minimize suffering.

### Isolation and culture of peritoneal mast cells

Cells were obtained by peritoneal lavage of male CD1 mice with HBSS (10 ml). RBCs were removed by osmotic lysis in distilled water, the cells returned to iso-osmolarity in HBSS (10×), and centrifuged (400×g, 5 min at 4°C). The resulting white cell pellet was washed once in HBSS by centrifugation (400×g, 5 min) and resuspended in 0.5 ml of 70% isotonic Percoll solution. The macrophage/monocyte layer was carefully removed before collection of the purified mast cell pellet. The purified mast cell preparations contained >98% mast cells, as determined by staining with toluidine blue. Cell viability was checked using the trypan blue exclusion method and cells were always >98% viable [16].

### Mast cell degranulation stains

Cells were plated in a 6-cm dish containing glass coverslips coated with poly-l-lysine (Sigma) in a 35-mm-diameter culture. For FcεRI-induced degranulation, P815 mast cells were sensitized by anti-IgE-DNP (Sigma, USA) overnight, and then stimulated by DNP-BSA (Invitrogen, USA) to induce degranulation. For compound 48/80 challenged degranulation, adding compound 48/80 (Sigma, USA) caused trigger degranulation. Mast cell degranulation morphometric staining was carried out by two dyes, toluidine blue and alcian blue.

### Mast cells activation for mediator release measurment

In compound 48/80 induced degranulation, cells were treated by 10 µg/ml compound 48/80. In IgE-mediated mast cell degranulation, cells were incubated with anti-IgE-DNP (Invitrogen) for 12 h before addition of 10 µg/ml DNP-BSA. Plates were incubated for 30 minutes and centrifuged, and the supernatant was decanted and stored at −20°C for the measurement of mediator content. Control cell pellets were lysed in ultrapure water for the determination of total histamine/tryptase content. Histamine was measured by OPT-fluorometric assay as reported previously [15]. In the fluorometric assay, histamine reacts with OPT to form a fluorophore. The fluorescent intensity was measured using the multifunctional microplate reader at λ_ex_ = 360 nm and λ_em_ = 460 nm, respectively. Tryptase-β2 (mMCP-6) was measured by measured by ELISA kit (R&D Systems) according to manufacture's instructions.

For the analysis of cytokine release, the final cell concentration was 1×10^6^ cells/ml, and cells were activated with LPS (final concentration 1 µg/ml, Sigma) for 24 hours. The cytokines TNF-α and IL-6 were measured by ELISA kit (R&D Systems) according to manufacture's instructions.

### Quantitative reverse transcription-polymerase chain reaction (RT-PCR)

Total RNA was extracted from cells with TRIzol reagent (Invitrogen). Quantitative RT-PCR was performed using 0.1 µg of cDNA with a SYBR Premix Ex Taq (Takara Bio, Shiga, Japan) and 10 pmol/µl primers. The primers used for real-time RT-PCR are: TNF-α: 5′-CAACCCTTAT TCTCGCTCACAA-3′ (forward) and 5′-GCCCACTTCTTTCCCTCACA-3′ (reverse), IL-6: 5′-GCTACCAAACTGGATATAATCAGGA-3′ (forward) and 5′-CCAGGTAGCTATGGTACTCCAGAA-3′ (reverse), glyceraldehyde-3-phosphate Dehydrogenase (GAPDH): 5′-CCAGCCTCGTCCCGTAGACA-3′ (forward), 5′-CCGTTGAATTTGCCGTGAGT-3′ (reverse). Reactions were run on a Stratagene Mx3000P (Agilent Technologies, Stockport, United Kingdom) real-time thermo cycler. Each sample was tested in triplicate. Results were analyzed with the ΔΔCt method using Mx3000P QPCR software.

### Immunofluorescence and confocal microscopy

Cultured cells on coverslips fixed with 4% paraformaldehyde for 15 min at room temperature. Following rinsing with PBS, cells were permeabilized with 0.3% Triton-X 100 (Sigma) for 15 min. When examined Tie-2 expression, this process should be avoided. After washing with PBS, the cells were blocked with normal horse serum for 1 h. Then incubated with primary antibodies for 24 h overnight. After rinsing with PBS, cells were incubated with fluorescein isothiocyanate (FITC)-conjugated goat anti-mouse IgG (1∶200) for 30 min at room temperature. Nuclei were counterstained with Hoechst 33342 (Sigma). Immunofluorescence was viewed under an Olympus FV1000 confocal laser scanning microscope.

### Western blotting

Total cellular protein and nucleic protein were prepared as previously described [17]. Protein concentration was determined using the BCA Protein Assay kit (Pierce). Proteins (30 µg) were subjected to 10% SDS polyacrylamide gel electrophoresis and subsequently transferred onto nitrocellulose membranes (Pall). After washing with 0.1% TBS-T, membranes were incubated for 1 h at room temperature in blocking buffer (5% nonfat milk in TBS-T) and then incubated with appropriate antibodies (1∶500 dilution, NF-κB from Cell Signaling, others are from Santa Cruz) overnight at 4°C. After washing with TBS-T, they were then incubated with the fluorescently-labeled secondary antibodies conjugated with Alexa Fluor 680 or IRdye 800 (1∶10000, Rockland Immunochemicals, Gilbertville, PA, USA) for 1 h at room temperature. The membranes were then washed with PBS containing 0.1% Tween-20 and scanned with the Odyssey Infrared Imaging System (LI-COR Biosciences, Lincoln, NE, USA) by setting the detection channels at 700 nm (for Alexa Fluor 680) and 800 nm (for IRDye 800CW). Scanned bands were quantified using the Odyssey software.

### Intracellular calcium imaging

After washing twice with phosphate buffer solution, the cells were incubated in the dark at 37°C in phosphate buffer solution containing 4 µM Fluo-3 acetoxymethyl ester. Approximately 20–30 min after stopping dye loading by refreshing the buffer, the dishes were transferred to an inverted microscope (Axiovert 100M, Leica) equipped with a 40× oil immersion objective (Leica, Plan-apochromatic, numerical aperture 0.65) in a CLSM system (Leica, TCS SP2, Germany). After a baseline scanning, stimulation agents added. Digital images (size 368.5×368.5 µm) were recorded (usually during 300 s) at RT with a spatial resolution of 512×512 pixels and a temporal resolution of one image per five seconds. The 488 nm argon laser line (200 mW) was used to excite Fluo-3 fluorescence in the cells, which was measured using a long-pass 543 nm filter. Laser illumination intensity was kept to a minimum (max 1% of laser output) to avoid phototoxicity and photobleaching.

Image data were analyzed off-line using the Leica TCS SP2 analyzing software. A selected image in each image set was used as a template for designating each cell. Because Fluo-3 is a single-wavelength indicator, it was not possible to apply the ratiometric method for quantitative determination of [Ca^2+^]_i_. Therefore, data were normalized with respect to the mean fluorescence intensity (Fo) during the first 30 s of recording. These relative fluorescence (RF) values represent integrated [Ca^2+^]_i_.

### Generation of lentivirus

The lentiviral vectors pWPI-IRES-EGFP (Addgene, USA) carried the elongation factor 1a (EF1a) promoter driving expression of enhanced green fluorescent protein (EGFP). Ang-1 cDNA (Regeneron Pharmaceuticals, USA) was inserted into the PmeI site upstream of the EMCV IRES under the control of EF1a promoter. Briefly, pWPI-IRES-EGFP, pMD2.G (Addgene, USA) and psPAX2 (Addgene, USA) were co-transfected into 293T cells and the resulting supernatant was collected after 24 h, 48 h, 72 h. The transfections were performed with lipofectamine 2000 reagent (Invitrogen). The supernatant was cleared of cell debris by filtering through a 0.45 µm filter. The eluted solution containing lentiviral vector was ultracentrifuged with a 20% (w/v) sucrose underlay for purification, and the infectious vector particle (titer) was determined in 293T cells. The titer was expressed as transducing units per milliliter (TU/ml) by using cell dilution method according to manufacture's guide.

### Measurement of Ang-1 Serum Levels in mice

Five-week-old mice were anesthetized with ketamine/xylazine and injected with 5×10^7^ TU of recombinant lentivirus-Ang-1 (LV-Ang-1) and lentivirus-EGFP (LV-GFP) vectors via the tail vein. For measurement of Ang-1 serum levels, mice were euthanized at time intervals 7, 14, 21, and 28 days after gene delivery (n = 5 each), inner canthus blood samples were obtained via capillary tubes. Serum Ang-1 was measured by measured by ELISA kit (R&D) according to manufacture's instructions.

### Compound 48/80-induced systemic anaphylaxis shock

Mice were given an intraperitoneal injection of 10 mg/kg body weight (BW) of compound 48/80. Mortality (%) within one hour was recorded (n = 10/group). After the mortality test, blood was obtained from the heart of each mouse to measure serum histamine and tryptase contents. For peritoneal lavage, 2 cm^2^ skin was removed leaving the peritoneal membrane intact. Then 1 ml of ice-cold 1× Hanks balanced salt solution (GIBCO) was injected using 26 gauge needle. After injection, peritoneum was gently palpated for 30 s, and peritoneal fluid was aspirated out using a 20 gauge needle. The fluid was centrifuged (600×g, 5 min), and the supernatant was stored at −20°C.

### Passive cutaneous anaphylaxis

An IgE-dependent cutaneous reaction was carried out as described previously [18]. The mice were injected intradermally in left ear with 0.5 mg of anti-DNP IgE. After 48 h, each mouse received an injection of 10 µg DNP-BSA containing 1% Evans blue via the tail vein. Thirty minutes after challenge, the mice were killed and the ears were removed for measurement of the amount of dye. The amount of dye was determined colorimetrically after extraction with 1 ml of 1 mM KOH and 9 ml of mixture of acetone and phosphoric acid (5∶13). The intensity of absorbance was measured at 620 nm in a spectrophotometer (Ultrospec 2100 pro, Amersham Biosciences, Cambridge.UK).

### Histological Analysis of Lung Tissue

Left lungs were inflated with 4% paraformaldehyde and fixed in the same fixative for 24 hours. Samples were processed and paraffin embedded. Serial sections were cut at 5 µm thickness and stained with hematoxylin and eosin (H&E). After examined by light microscopy for evidence of lung injury, the lung injury was scored for edema, neutrophil infiltration, hemorrhage [19]. A score scaled at 0 to 4 represents the severity: 0 for no or very minor, 1 for modest and limited, 2 for intermediate, 3 for widespread or prominent, and 4 for widespread and most prominent.

### Mesentery mast cell stains

The mesentery was removed from animals along with the intestines and placed in Petri dishes whose bottoms were covered with paraffin. The mesenteries were stretched over the paraffin and secured with fine needles. The mesentery was then fixed in 4% paraformaldehyde in PBS and stained for 15 min in 0.1% Toluidine blue-1% acetic acid, pH 2.8. Mesentery fragments were stretched over glass slides, dried on a heating plate at 45°C, dehydrated in a graded series of ethanol (Merck), cleared in xylene (Merck), and coverslips mounted with Permount (Fisher Scientific, Atlanta, GA).

### Statistical analysis

Data were statistically analyzed using a one-way ANOVA test or Student's *t*-test. *P*-values less than 0.05 were considered statistically significant.

## Results

### Ang-1 inhibited LPS-induced pro-inflammatory cytokines release of mast cells by suppressing IκB phosphorylation and NF-κB nuclear translocation

**Figure 1:**
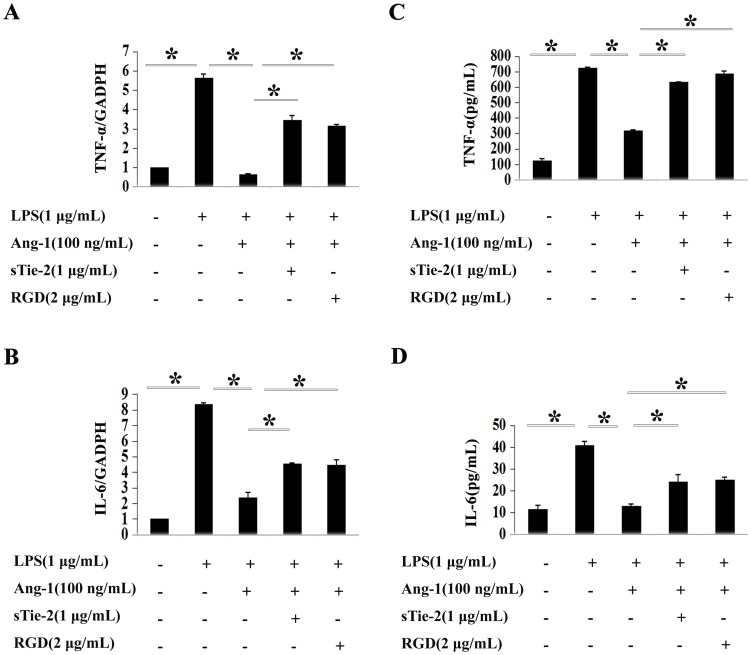
Ang-1 inhibited LPS-induced cytokines production in P815 mast cells. Quantitative RT-PCR (A and B) and ELISA (C and D) were employed for detection of mRNA and the secretion of cytokines production in duplicates, respectively. **A and C:** LPS dramatically increased TNF-α mRNA production and protein secretion in P815 mast cells (column 2 versus column 1). Addition of Ang-1 abrogated the induction of LPS on mast cells (column 3 versus column 2). Soluble form of Tie2 (sTie-2) and RGD reversed the inhibition of Ang-1 on LPS-induced TNF-α production (column 4, 5 versus column 3). *P<0.05. **B and D:** IL-6 mRNA and secretion were both significantly increased in mast cells upon LPS treatment. Ang-1 could decrease the induction while sTie-2 and RGD exerts opposite effects. *P<0.05.

TLRs (Toll like receptors) are critical in innate and acquired immunity [20]. TLR activation on mast cells leads to the release of different cytokines, such as TNF-α and IL-6 [21]. P815 mast cells express TLR4, which can be triggered by LPS [22]. After incubation mast cells for 4 h with LPS, real-time PCR assay showed LPS stimulated TNF-α mRNA expression level increasing around five times that of the control group, and Ang-1 inhibited the elevation (Figure 1A). Real-time PCR were used to examine IL-6 mRNA expression levels in LPS-challenged P815 mast cells for 2 h, we got the same results (Figure 1B). In parallel experiments, ELISA revealed the same effects of Ang-1 on TNF-α and IL-6 secretion (Figure 1C and 1D).

**Figure 2:**
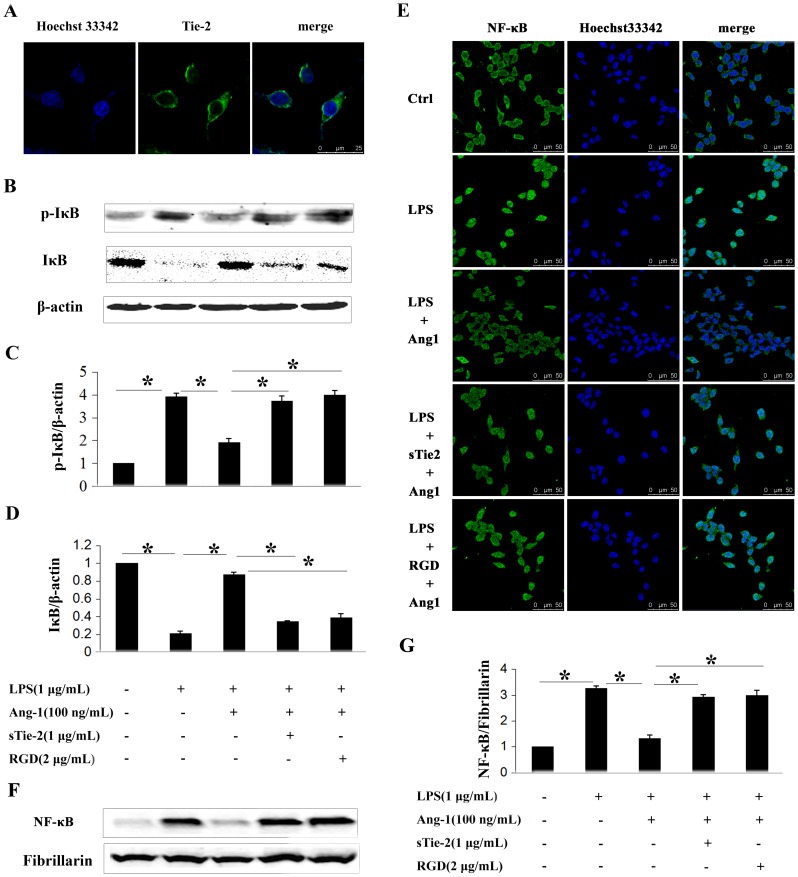
Ang-1 inhibited LPS-induced IκB phosphorylation and NF-κB nuclear translocation in P815 mast cells. **A:** Immunofluorescence showed Tie-2 receptor expression. Primary antibody in negative control was monoclonal IgG. **B:** Western blotting was performed to analyze phosphorylation levels of IκB in mast cells in response to different stimuli. **C and D:** Densitometric analysis was used to calculate the relative ratio of p-IκB/β-actin (C) and IκB/β-actin (D). Ratio of the control group was arbitrarily presented as 1. *P<0.05. **E:** NF-κB translocation was detected by confocal microscopy. In quiescent P815 mast cells, NF-κB exerts primarily in the cytoplasm (first panel). With LPS treatment for 2 h, NF-κB translocates into the nucleus (second panel). Ang-1 nearly abolished LPS induced translocation of NF-κB (third panel) which was reversed by soluble form of Tie2 (sTie-2) (forth panel) and RGD (fifth panel). **F:** NF-κB translocation was also detected by Western blot. Fibrillarin was used as internal control. **G:** Densitometric analysis was used to calculate the relative ratio of NF-κB/Fibrillarin. Ratio of the control group was arbitrarily presented as 1. Data are mean ± SD of the ratios from 3 independent experiments. *P<0.05.

Results from immunofluorescence staining revealed that P815 mast cells express Tie-2 receptor *in vitro*, suggesting a role of the Ang-1/Tie-2 system in mast cell activation (Figure 2A). Ang-1 has also been shown to mediate cellular functions in endothelial and non-endothelial cells via direct binding and interaction with integrins [23], [24], [25]. For this reason, P815 mast cells were pre-incubated with RGD peptides or sTie-2 (soluble extracellular fragment of Tie-2 and Fc fusion protein) to abrogate integrin and Tie-2 activation by Ang-1, respectively.

We first simultaneously added 1 µg/ml sTie-2 and Ang-1 to culture P815 mast cells before challenging the cells with LPS. The results suggested that sTie-2 reversed the inhibitive functions of Ang-1 on both mRNA and protein levels of TNF-α (P<0.05) and IL-6 (P<0.05) (Figure 1A–D). The addition of RGD, which blocked the integrin pathway, showed that the function of Ang-1 on LPS-induced TNF-α and IL-6 production of mast cells was also abrogated (Figure 1A–D). Furthermore, we repeated all the above tests using primary cultured mouse peritoneal mast cells, and got similar results (Figure S1).

We further explored the mechanism through which Ang-1 inhibited LPS-induced activation of mast cells. NF-κB is an important transcript factor that controls the expression of many pro-inflammatory cytokines [26]. LPS can phosphorylate IκB, which is an inhibitor of NF-κB. Phosphorylation targets IκB for rapid degradation through the ubiquitin-proteasome pathway, allowing the nuclear translocation of NF-κB. In the present study, Western blot analysis demonstrated that LPS phosphorylated IκB and decreased the total amount of IκB (Figure 2B–D). With administration of Ang-1 prior to LPS challenge, the effect was inhibited. Furthermore, with the addition of sTie-2 or RGD, the inhibitive role of Ang-1 was alleviated (Figure 2B–D).

We also examined LPS-induced nuclear translocation of NF-κB by immunofluorescence assay (Figure 2E) and western blotting analysis (Figure 2F–G). NF-κB existed primarily in the cytoplasm, and was translocated to the nucleus after LPS treatment. Immunofluorescence revealed the same that Ang-1 inhibited nuclear translocation of NF-κB, and the effect could be reversed by sTie-2 or RGD (Figure 2E). Western blotting assay also demonstrated the same phenomena (Figure 2F–G).

### Ang-1 protected against mast cell degranulation and inflammatory mediators release through decreasing intracellular calcium levels

**Figure 3:**
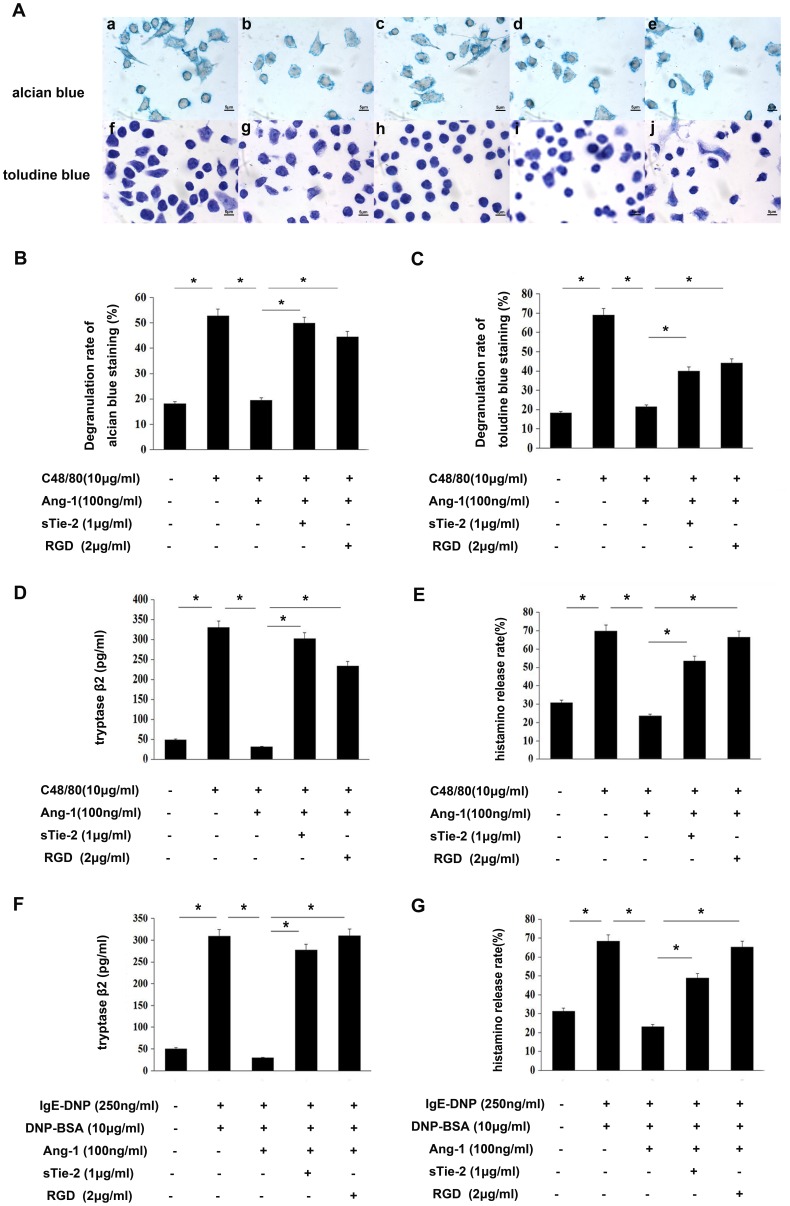
Ang-1 suppressed compound 48/80 induced mast cells degranulation. Degranulation was determined by staining with dyes and measuring the release of histamine and tryptase. **A:** Mast cells degranulation was observed by microscope 20 min after compound 48/80 10 µg/mL treatment. Cells were stained with alcian blue (a–e) and toluidine blue (f–j) (100×). (a, f) control group; (b, g) compound 48/80-treated cells; (c, h) Ang-1 100 ng/ml-treated cells; (d, i) Soluble form of Tie2 (sTie-2)-treated cells and (e, j) RGD-treated cells. **B and C:** Quantification of P815 mast cells degranulation by compound 48/80. It was performed in a blinded fashion. **D:** Degranulation stimulated by compound 48/80 was determined by measuring the release of tryptaseβ2 (mMCP-6) through commercial ELISA kit in duplicates. The data shown are mean±SD of 3 separate experiments. **E:** Degranulation stimulated by compound 48/80 was determined by measuring the release of histamine through OPT-fluorometric assay as previously reported in duplicates. **F:** Mast cells degranulation were incubated with 250 ng/ml before DNP-BSA 10 µg/ml treatment for 20 min. Degranulation was determined by measuring the release of tryptase-β2 (mMCP-6) through commercial ELISA kit in duplicates. The data shown are mean ±SD of 3 separate experiments. **G:** Mast cells degranulation were incubated with 250 ng/ml before DNP-BSA 10 µg/ml treatment for 20 min. Degranulation was determined by measuring the release of histamine through OPT-fluorometric assay as previously reported in duplicates. *P<0.05.

Compound 48/80 is a potent basic secretagogue that can induce mast cell degranulation and mediator release. We used 10 µg/ml of compound 48/80 to induce P815 mast cells and peritoneal mast cell degranulation. After staining with toluidine blue, glass slides were examined by bright field microscopy and the percentages of degranulated mast cells were determined (Figure 3A). Incubation with phosphate buffered saline (PBS) in the control group showed approximately 18.44%±6.54% basic degranulation, and incubation with compound 48/80 resulted in approximately 57.23%±10.21% degranulation. The degranulation rate decreased to 21.45%±1.26% with the administration of Ang-1. The addition of sTie-2 or RGD blocked Tie-2 or integrin receptor, respectively, and abrogated the inhibitive function of Ang-1 (Figure 3B and 3C).

We also examined histamine and tryptaseβ2 release. Results demonstrated that Ang-1 decreased histamine and tryptaseβ2 release levels, respectively, and this function could be abrogated by sTie-2 or RGD (Figure 3D and 3E). Furthermore, we repeated all the above tests using primary cultured mouse peritoneal mast cells, and got the similar results (Figure S2).

It was well known that when the high-affinity IgE receptors (FcεRI) on mast cells were cross-linking induced by allergens,it results in degranulation. So we repeated our experiments using IgE-DNP/DNP-BSA stimuli, and got similar results (Figure 3F–G, Figure S3).

**Figure 4:**
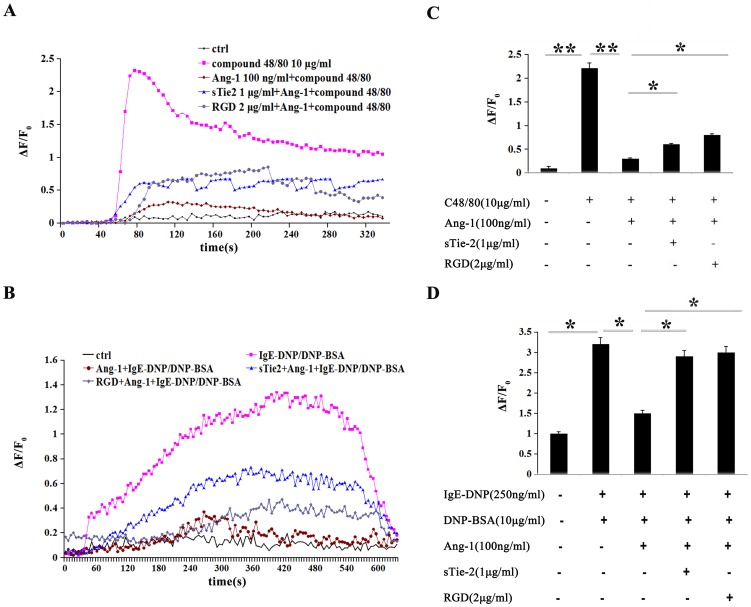
Ang-1 suppressed compound 48/80 stimulated and FcεRI-mediated Ca^2+^ mobilization. **A and B:** Ca^2+^ response to compound 48/80 (A) or IgE-DNP/DNP-BSA (B) activation in control, Ang-1-treated, sTie2-treated and RGD treated cells. **C and D:** Statistical analysis of the amplitude of the compound 48/80 stimulated (C) and FcεRI-induced (D) Ca^2+^ increase in both groups. The amplitude of the Ca^2+^ response was represented as the highest observed level of ΔF/F_0_. Data indicate mean ± SD from four independent experiments. *P<0.05,**P<0.001.

Given the key role of intracellular Ca^2+^ signaling in mast cell degranulation, we explored any possible modulating effects of Ang-1 on compound 48/80-induced Ca^2+^ mobilization in mast cells. After P815 mast cells were loaded with Fluo-3/AM, we measured the [Ca^2+^]i response to bath application of compound 48/80. Calcium images of mast cells were measured (five images/s) during a period of 3–4 min. Application of the secretagogue compound 48/80 (10 µg/ml) induced a complex increase in [Ca^2+^]i in mast cells (Figure 4A). Along with inhibiting mast cell degranulation, Ang-1 treatment (100 ng/ml) significantly decreased the peak amplitude of the Ca^2+^ increase (indexed by the highest level of ΔF/F0) by 86.45%±2.53%, from 2.3±0.06 in the challenged group (n = 16) to 0.31±0.05 in the Ang-1-treated group (n = 16) (*P*<0.01) (Figure 4C). This result indicated that Ang-1 may suppress mast cell degranulation trigged by compound 48/80 through inhibition of intracellular Ca^2+^ mobilization. We also explored Ang-1's function in FcεRI-mediated mast cell degranulation and got the same results (Figure 4B and 4D).

These results above suggest that Ang-1 may play a role in suppressing intracellular Ca^2+^ mobilization, then resulting in inhibitive function on degranulation.

### Ang-1 alleviated passive cutaneous anaphylaxis reaction in vivo

**Figure 5:**
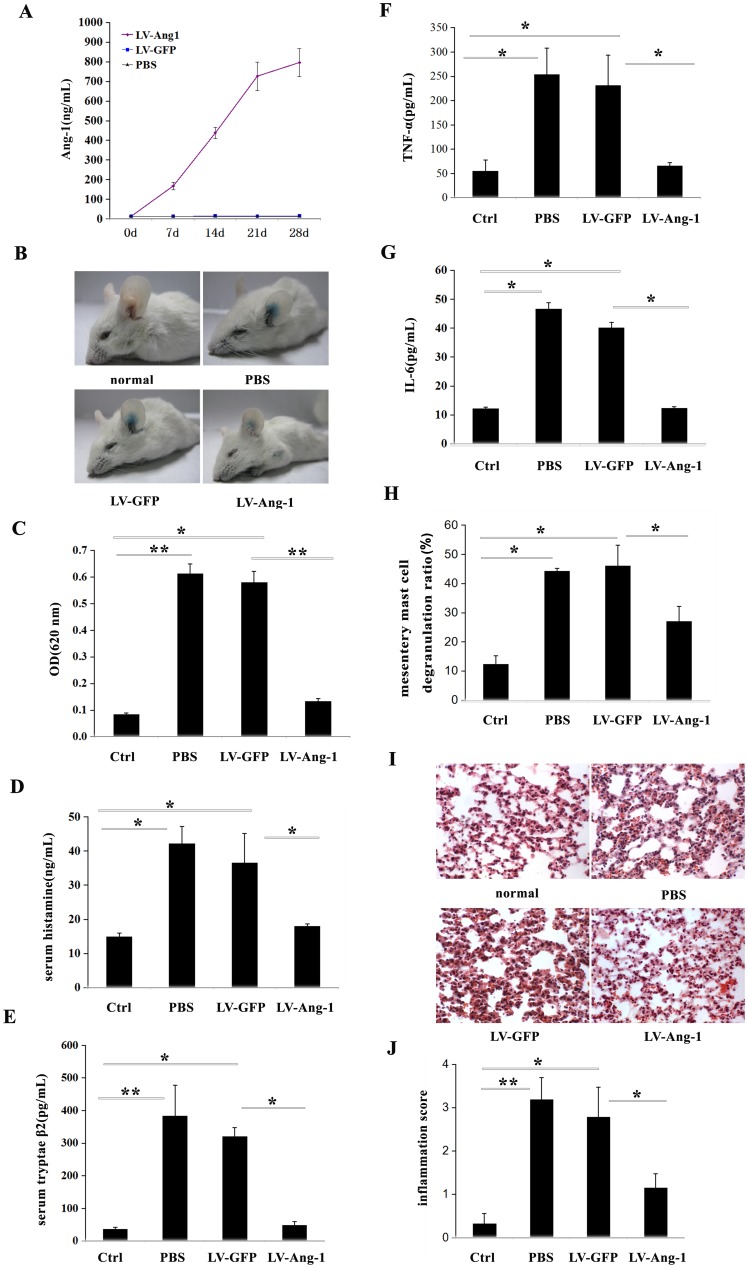
Ectopic expression of Ang-1 *in vivo* protected against IgE-dependent PCA and anaphylaxis shock. **A:** The measurements of serum Ang-1 levels by ELISA in 7, 14, 21 and 28 days after intravenous injection of 1×10^6^ TU LV-Ang-1. Each data point indicates mean±SD of 5 mice. **B and C:** The anti-allergic effect was firstly assessed using IgE-DNP/DNP-BSA induced PCA mouse model. PCA mice showed ear Evans blue exudation (B), and the absorbance value was detected (C). **D:** Serum histamine of systemic anaphylaxis shock mice was determined through OPT-fluorometric assay as previously reported. Each data point indicates mean ±SD of 6 mice. **E:** ELISA kit detected serum tryptase-β2 of systemic anaphylaxis shock mices. Each data point indicates mean±SD of 6 mice. **F and G:** Peritoneal TNF-α and IL-6 levels in systemic anaphylaxis shock mices were detected by ELISA. Data indicate mean±SD from four independent experiments. **H:** In compound 48/80 induced systemic anaphylaxis shock model,mesentery mast cells degranulation was detected by toluidine blue staining. Quantification of mesentery mast cells degranulation by compound 48/80 was performed in a blinded fashion. **I:** Lung injury was detected by hematoxylin and eosin staining in systemic anaphylaxis shock mices. **J:** Statistical analysis of the lung injury score in systemic anaphylaxis shock mices by assessment infiltration of numerous polymorphonuclear leukocytes, interstitial spaces and hemorrhage, as described in materials and methods. It was performed in a blinded fashion. Each data point indicates mean±SD of 5 mice. *P<0.05.

The findings that Ang-1 had the capacity to stabilize mast cells *in vitro* suggested that Ang-1 might have a therapeutic effect during allergic diseases. To clarifythis hypothesis, we ascertained the effects of Ang-1 treatment using an *in vivo* model of mast cell-dependent passive cutaneous anaphylaxis (PCA) mice. A lentivirus construct encoding Ang-1 was administered by intravenous injection. Ang-1 protein expression concentrations in serum were measured using ELISA. After 2 weeks, the serum Ang-1 concentration was 492 ng/ml, and remained elevated for at least 4 weeks (Figure 5A). Consistent with previous reports, antigen stimulation markedly increased vascular permeability, as indicated by the amount of Evans blue dye extravasation (Figure 5B and 5C). To evaluate permeability, the absorbance value of Evans blue dye was evaluated. The control group without Ang-1 pretreatment showed more severe dye extravasation than the Ang-1 treatment group, with an absorbance value of 0.58±0.05 to 0.13±0.03 (Figure 5C). The results suggested that Ang-1 treatment inhibited IgE-dependent PCA.

### Ang-1 improved survival in compound 48/80-induced systemic anaphylaxis

Since the 1950s, compound 48/80 has been used to establish animal anaphylaxis shock models. Compound 48/80-treated mice showed signs of severe systemic anaphylaxis, such as reduced mobility, apathy, conjunctivitis, and fur ruffling, within a few minutes of administration. Ang-1-lentivirus-treated challenged mice exhibited fewer signs of sickness than control mice.

To investigate whether Ang-1 gene transfer could improve survival in compound 48/80-induced lethal anaphylaxis shock, the mouse mortality rate over 1 h was examined. Within 1 h after compound 48/80 injection, all challenged control mice, but only 50% of Ang-1-lentivirus-treated mice had died (Table 1). The results suggest that lentivirus over-expression of Ang-1 in mice significantly increased survival from compound 48/80-induced anaphylaxis shock.

**Table 1:** Effects of Ang-1 on compound 48/80-induced systemic anaphylactic shock.

Group	Number of lentivius (TU)	Compound 48/80	mortality
**Normal**	0	−	0
**PBS**	0	+	100%
**LV-GFP**	5×10^6^	+	100%
**LV-Ang-1**	5×10^6^	+	50%*

Groups of mice (n = 10/group) were intravenously administrated with PBS, LV-GFP or LV-Ang-1 28 days before the intraperitoneal injection of compound 48/80. Mortality (%) within 1 h following compound 48/80 injection is represented as the number of dead mice×100/total number of experimental mice.

*P<0.05.

Examination of serum histamine and tryptase showed decreased release levels in the Ang-1-lentivirus-treated group (Figure 5D and 5E). Examination of TNF-α and IL-6 protein expression, revealed very low expression levels of both pro-inflammatory molecules in the peritoneal fluid of normal control mice (Figure 5F and 5G). Systemic anaphylaxis challenge by compound 48/80 substantially increased the expression of peritoneal fluid TNF-α and IL-6 proteins. In contrast, expression levels of both proteins in Ang-1-lentivirus-treated mice were attenuated compared with PBS or GFP-lentivirus pretreated mice (Figure 5F and 5G). Toluidine blue staining of mesentery membranes showed that mast cell degranulation had improved in the Ang-1-lentivirus-treated group (Figure 5H). Light microscopy of lungs taken from the anaphylaxis shock model mice showed an infiltration of numerous polymorphonuclear leukocytes and macrophages into the interstitial spaces, hemorrhaging, and marked swelling of the alveolar walls. These changes were all attenuated in systemic anaphylaxis mice pretreated with Ang-1-lentivirus (Figure 5I and 5J).

## Discussion

Mast cells play an important role in inflammation and allergy diseases. The cytoplasm of mast cells contains organelles-lipid bodies where metabolism of arachidonic acid occurs and where the products of this metabolism, including leukotrienes, are stored [27]. Cytokines and histamine are products found in mast cells organelles.

The present study used three irritant agents: LPS, compound 48/80, and IgE-DNP/DNP-BSA. The results suggest that Ang-1 may exhibit protective roles on mast cell activation, possibly through Tie-2 or integrin. The results also demonstrate that bacterial LPS binds to TLR4 on the P815 mast cell surface, and activates the IκB/NF-κB pathway to regulate TNF-α and IL-6 expression levels. LPS-induced mast cell activation was characterized by differential inflammation release without degranulation. Although LPS failed to have a direct effect on mast cell degranulation, it did appear to enhance degranulation in RBL-2H3 cells and mouse peritoneal mast cells upon FcεRI activation [28]. Reports demonstrate that Ang-1 inhibits NF-κB activation through interacting with NF-κB inhibitor ABIN-2 [29]. In the present study, Ang-1 inhibited LPS-induced activation of NF-κB, regulating TNF-α and IL-6 expression levels. Further study is required to determine whether ABIN-2 is involved.

Mast cell degranulation, challenged with compound 48/80 or IgE-DNP/DNP-BSA, showed elevated intracellular calcium levels in the present study. It is well known that intracellular calcium elevation is crucial in mast cell degranulation. However, the Ca^2+^ entry pathway has not been identified. It is reported that store operate calcium entry is the main mode of Ca^2+^ entry in intracellular calcium elevation [30]. The best characterized channels are the calcium release activated channels (CRAC). Ashmole *et al.* suggested that the CRACM/Orai ion channel family may be the underlying channels for influx of extracellular Ca^2+^ into human lung mast cells [31]. It is suggested that the L-type calcium channel may participate in regulation of store-operated channels in murine BMMC and RBL-2H3 mast cells [32], [33]. It has also been suggested that the non selective transient receptor potential canonical (TRPC) cation channels are necessary for FcεRI-mediated full Ca^2+^ influx and degranulation in BMMC and RBL-2H3 cells [32]. Researchers have demonstrated that Ang-1 blocks TRPC1-dependent Ca^2+^ influx induced by VEGF by interfering with interactions between IP_3_R and TRPC1, thereby abrogating increases in endothelial permeability [33]. Therefore, Ang-1 may regulate intracellular calcium elevation in mast cell degranulation through the inhibition of TRPC1.

As it has been reported that Ang-1 could bind to Tie-2 to regulate macrophage activation, we examined whether Tie-2 was involved in the effective functions of Ang-1 on mast cells. Integrins are known to cooperate with growth factors to regulate several cellular functions. Many cellular responses to soluble growth factors, such as epidermal growth factor and platelet-derived growth factor, depend on the cell adhering to a substrate via integrins. This is the essence of anchorage-dependent cell survival and proliferation, and integrins form the basis of these phenomena [34]. Cross-talk between integrins and growth factor receptors were shown to coordinate biologic processes through the regulation of downstream and inside-out signaling pathways [35], [36]. It has been demonstrated in endothelial cells that Ang-1/Tie-2 activates integrins through PI3-K signaling, which suggests that FAK recruitment to the Tie-2/α5β1 complex could be dependent on activated Tie-2 inside-out signaling [37]. By modulating the adhesive properties of glioma cells, and, in particular, by increasing integrin β1 expression, and by activating integrin β1 signaling, Ang-1/Tie-2 might contribute to the increased aggressive behavior of human brain tumors [38]. In the present study, Ang-1 activated both Tie-2 and integrin to modulate mast cell activation. This may suggest cross-talk between Tie-2 and integrin.

Anaphylaxis is a life-threatening syndrome induced by a sudden systemic release of inflammatory mediators, such as histamine, tryptase, various cytokines, and lipid-derived mediators [39]. Using a compound 48/80-induced murine model, we observed inhibitive functions of Ang-1 in anaphylaxis shock *in vivo*. Consistent with *in vitro* results, Ang-1 over-expression showed an inhibitive role on serum histamine and tryptase release levels. We observed that mast cells accumulated in the peritoneal cavity after compound 48/80 injection, and found that Ang-1 alleviated mesentery mast cell degranulation.

Cross-linking of FcεRI can lead to an increase in TNF-α mRNA expression. Newly synthesized TNF-α, which amplifies the allergic symptoms by inducing chemotaxis of neutrophils and other inflammatory cytokine production, plays a critical role in the late phase of an anaphylaxis reaction [40]. IL-6 is also a pro-inflammatory cytokine, correlates with the extent of erythema, is inversely related to mean arterial pressure, correlates strongly with the occurrence of hypotension, causes increased expression of FcεRI and increased intracellular histamine, and prevents mast cell apoptosis [39]. The levels of TNF-α and IL-6 in peritoneal fluid decreased in the Ang-1-treated mice anaphylaxis model.

As a target organ for inflammation and allergy, the lung is also abundant in mast cells. Ang-1 showed a protective role on lung function in endotoxic shock and asthma. We examined inflammation in lung sections, and observed that Ang-1 over-expression could alleviate lung injury in the murine anaphylaxis shock model. Vessel exudation is one feature in anaphylaxis. The *in vivo* experiments using the passive cutaneous model showed that Ang-1 over-expression could decrease vessel permeability.

Studies have examined the effects of Ang-1 and have revealed its potent functions in many activities, such as angiogenesis [1], stem cell niche regulation [40], [41], and vessel permeability [4]. Recently, Ang-1 has shown a direct function on neutrophils [9] and macrophages [11]. In the present study, we observed that Ang-1 could inhibit the activation of mast cells, and positively treat anaphylaxis in murine models. These results aid in understanding the effects of Ang-1, and may contribute to further research and the prospects of Ang-1 being used for clinical treatment.

## Supporting Information

Figure S1 **Ang-1 inhibited LPS-induced peritoneal mast cells activation.** Quantitative RT-PCR (A and B) and ELISA (C and D) were employed for detection of mRNA and the secretion of cytokines production in duplicates, respectively. **A and C:** LPS dramatically increased TNF-α mRNA production and protein secretion in peritoneal mast cells (column 2 versus column 1). Addition of Ang-1 abrogated the induction of LPS on mast cells (column 3 versus column 2). Soluble form of Tie2 (sTie-2) and RGD reversed the inhibition of Ang-1 on LPS-induced TNF-α prodution (column 4,5 versus column 3). *P<0.05. **B and D:** IL-6 mRNA and secretion were both significantly increased in mast cells upon LPS treatment. Ang-l could decrease the induction while sTie-2 and RGD exerts opposite effects. *P<0.05.
(TIF)

Figure S2 **Ang-1 suppressed compound 48/80-induced peritoneal mast cells degranulation.** Mast cell degranulation was assessed using specific stains and measured by the relative release of histamine and tryptase. **A:** toluidine blue staining of peritoneal mast cells. (a) control group; (b) compound 48/80-treated cells; (c) Ang-1 100 ng/ml-treated cells; (d) Soluble form of Tie2 (sTie-2)-treated cells and (e) RGD-treated cells. **B:** Statistical analysis of amplitudes of compound 48/80-induced cell degranulation from all groups. It was performed in a blinded fashion. The data shown is the mean±SD of 3 separate experiments. **C:** Degranulation stimulated by compound 48/80 was determined by measuring the release of histamine through OPT-fluorometric assay as previously reported in duplicates. **D:** Degranulation stimulated by compound 48/80 was determined by measuring the release of tryptase-β2 (mMCP-6) through commercial ELISA kit in duplicates. The data shown are mean ±SD of 3 separate experiments. *P<0.05.
(TIF)

Figure S3 **Ang-1 suppressed FcεRI-mediated mast cells degranulation.** Degranulation was determined by staining with dyes and measuring the release of histamine and trptase. **A:** Mast cell degranulation was observed by microscope 20 min after DNP-BSA 10 µg/ml treatment after overnight incubated with 250 ng/ml. Cells were stained with alcian blue (a–e) and toluidine blue (f–j) (100×). (a,f) control group, (b,g) IgE-DNP/DNP-BSA-treated cells, (c,h) Ang-1 100 ng/ml-treated cells, (d,i) Soluble form of Tie2 (sTie-2)-treated cells and (e,j) RGD-treated cells. **B and C:** Quantification of P815 mast cells degranulation by IgE-DNP/DNP-BSA. It was performed in a blinded fashion. The data shown is the mean±SD of 3 separate experiments. *P<0.05.
(TIF)
