# Supplementary figures and images for: Angiopoietin1 Inhibits Mast Cell Activation and Protects against Anaphylaxis
Source: PLoS One. 2014 Feb 19;9(2):e89148. doi: 10.1371/journal.pone.0089148 (PMC3929638; doi:10.1371/journal.pone.0089148)

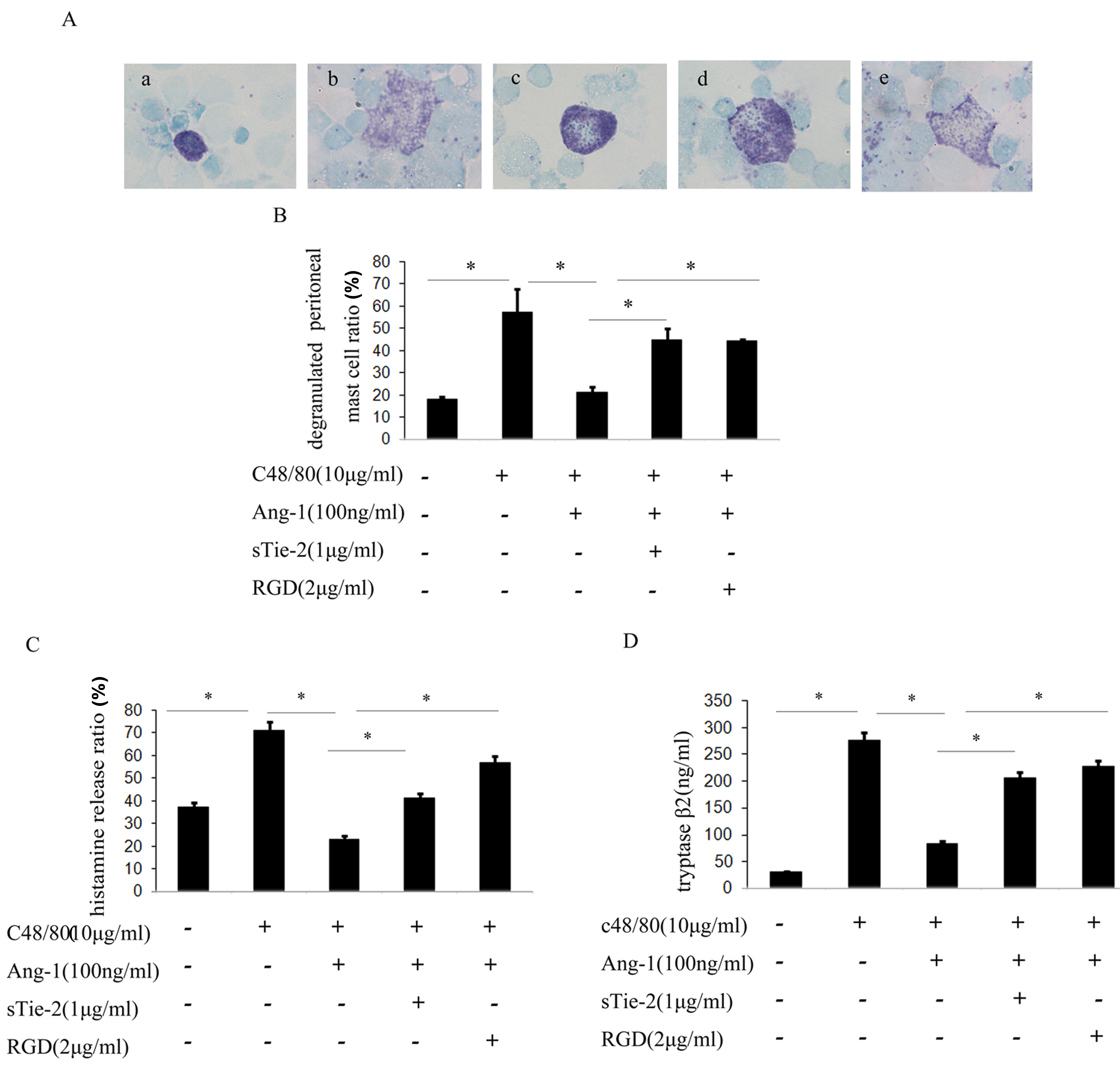

Supplement: Figure S2 — Ang-1 suppressed compound 48/80-induced peritoneal mast cells degranulation. Mast cell degranulation was assessed using specific stains and measured by the relative release of histamine and tryptase. A: toluidine blue staining of peritoneal mast cells. (a) control group; (b) compound 48/80-treated cells; (c) Ang-1 100 ng/ml-treated cells; (d) Soluble form of Tie2 (sTie-2)-treated cells and (e) RGD-treated cells. B: Statistical analysis of amplitudes of compound 48/80-induced cell degranulation from all groups. It was performed in a blinded fashion. The data shown is the mean±SD of 3 separate experiments. C: Degranulation stimulated by compound 48/80 was determined by measuring the release of histamine through OPT-fluorometric assay as previously reported in duplicates. D: Degranulation stimulated by compound 48/80 was determined by measuring the release of tryptase-β2 (mMCP-6) through commercial ELISA kit in duplicates. The data shown are mean ±SD of 3 separate experiments. *P<0.05. (TIF) [file pone.0089148.s002.tif]

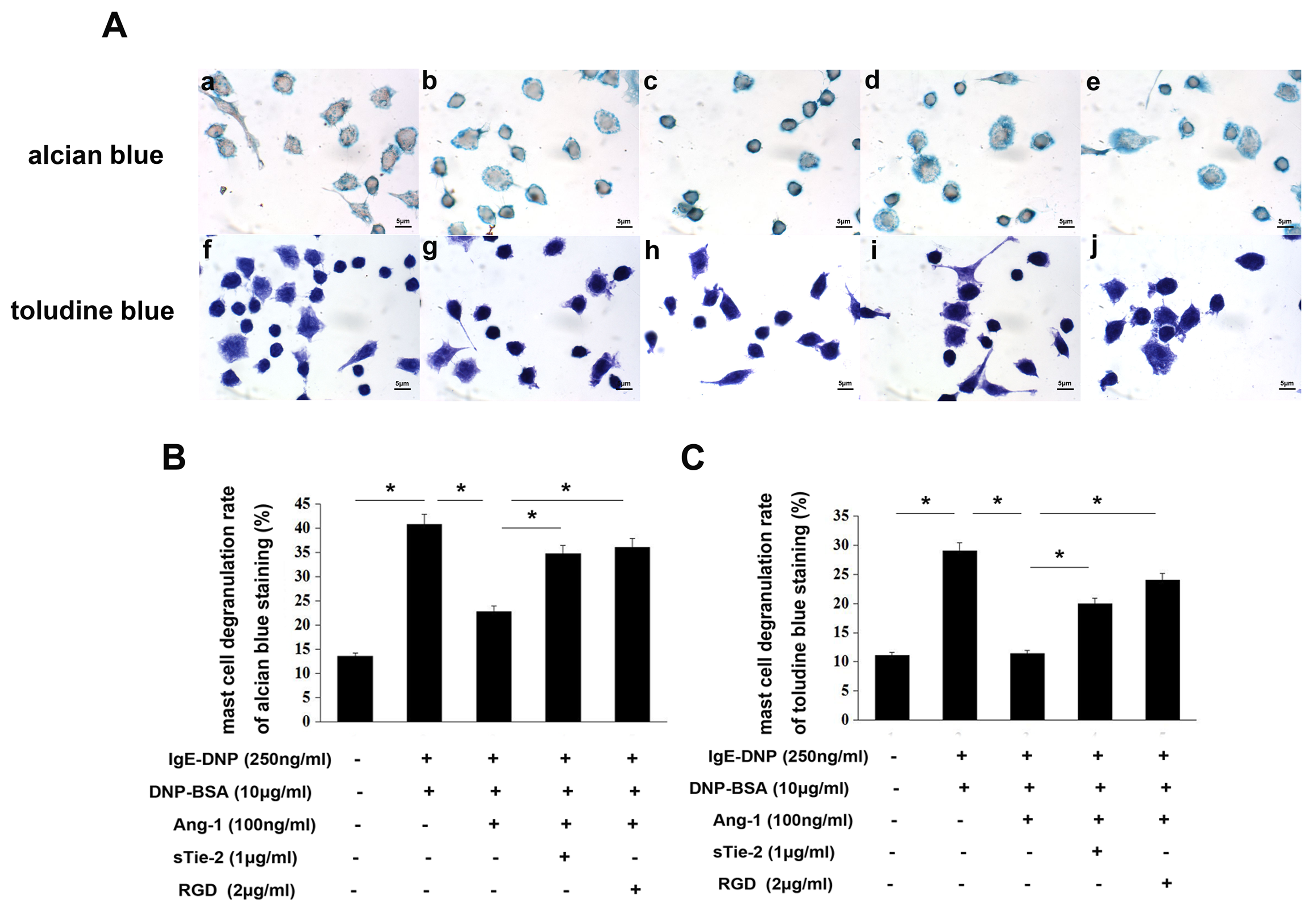

Supplement: Figure S3 — Ang-1 suppressed FcεRI-mediated mast cells degranulation. Degranulation was determined by staining with dyes and measuring the release of histamine and trptase. A: Mast cell degranulation was observed by microscope 20 min after DNP-BSA 10 µg/ml treatment after overnight incubated with 250 ng/ml. Cells were stained with alcian blue (a–e) and toluidine blue (f–j) (100×). (a,f) control group, (b,g) IgE-DNP/DNP-BSA-treated cells, (c,h) Ang-1 100 ng/ml-treated cells, (d,i) Soluble form of Tie2 (sTie-2)-treated cells and (e,j) RGD-treated cells. B and C: Quantification of P815 mast cells degranulation by IgE-DNP/DNP-BSA. It was performed in a blinded fashion. The data shown is the mean±SD of 3 separate experiments. *P<0.05. (TIF) [file pone.0089148.s003.tif]
